# Mechanical Prophylactic Measures against Influenza and Some Other Infectious Diseases

**Published:** 1918-11

**Authors:** 


***Mechanical Prophylactic Measures Against Influenza and some other Infectious Diseases.*** By MM. VINCENT and LOCHON. *La Presse Médicale*, October 17, 1918.

The spreading of influenza is caused by direct contact with patients, and through soiled linen, but most of all by means of the contaminated atmosphere surrounding every patient.

Each of these is surrounded by an infected atmosphere constituted by small particles of sputum, mucus, or pus containing great numbers of pathogenic organisms.

On placing Pétri boxes, with agar slides, within 20 cm., 50 cm., or 1 meter from the mouth of a patient who talks, coughs, or sneezes, it is amazing to see how many strains of bacteria develop subsequently on the nutrient medium. After 2 minutes'talk, the culture shows 209 strains; 3 or 4 fits of coughing develop 300 to 650 strains.

The virus of influenza (just like that of whooping-cough, measles, meningitis, diphtheritis, mumps) grows at first on the conjunctivae, in the nasal fossae, pharynx and larynx.

The position of the patients is quite comparable to that of soldiers surrounded by an atmosphere of "ypérit" gas. Similar modes of protection have seemed advisable in both cases.

American doctors make use in their wards of gauze compresses placed on nose and mouth; but it appears necessary to protect the eyes also, since they are one of the possible passages for infection.

The authors have experimented what thickness of gauze is really effective to stop the morbid germs — patients have been made to speak, cough, sneeze in front of agar slides protected by one, two, or three ... gauzes; 5 sheets of gauze presenting 10 threads per cm. give efficient protection. The cultures tried in that case show but very few strains.

The authors present a " cagoule " devised according to this scheme, transparent in front of the eyes. It may be placed directly upon the head, or put on a light metallic frame.

This mask proves simple, cheap, easy to sterilize or to change.

